# Plasma ubiquitin C-terminal hydrolase L1 levels reflect disease stage and motor severity in Parkinson’s disease

**DOI:** 10.18632/aging.102695

**Published:** 2020-01-13

**Authors:** Adeline Su Lyn Ng, Yi Jayne Tan, Zhonghao Lu, Ebonne Yulin Ng, Samuel Yong Ern Ng, Nicole Shuang Yu Chia, Fiona Setiawan, Zheyu Xu, Nicole Chwee Har Keong, Kay Yaw Tay, Wing Lok Au, Louis Chew Seng Tan, Eng-King Tan

**Affiliations:** 1 Department of Neurology, National Neuroscience Institute, Tan Tock Seng Hospital, 308433, Singapore; 2 Neuroscience and Behavioural Disorders Program, Duke-NUS Medical School, 169857, Singapore; 3 Department of Neurology, National Neuroscience Institute, Singapore General Hospital, 169856, Singapore; 4 Department of Neurosurgery, National Neuroscience Institute, Tan Tock Seng Hospital, 308433, Singapore

**Keywords:** Parkinson’s disease, ubiquitin, UCHL1, biomarkers, blood

## Abstract

Parkinson’s disease (PD) is characterized by Lewy bodies containing α-synuclein and ubiquitin aggregates, their co-occurrence possibly linked to a failure of the ubiquitin proteasome system. Ubiquitin C-terminal hydrolase L1 (UCHL1) plays an important role in maintenance of nervous system integrity, and overexpression of UCHL1 has been shown to increase ubiquitin levels within neurons. While cerebrospinal fluid ubiquitin levels were reported to be lower in PD vs controls, plasma UCHL1 levels and their relationship with clinical measures in PD has not been reported. We measured plasma UCHL1 levels using single molecule array (Simoa) in 291 subjects (242 PD and 49 healthy controls, HC). We found that UCHL1 levels were significantly higher in PD patients at moderate stages (Hoehn and Yahr, H&Y stage >2) vs milder PD (H&Y ≤2, *p*<0.001) and HC (*p*=0.001). There was no significant difference in UCHL1 levels between PD patients at H&Y stages ≤2 vs HC. Across all PD patients, UCHL1 correlated significantly with UPDRS Part III motor scores (*β*=3.87, 95% CI=0.43-7.31, *p*=0.028), but not with global cognition. Overall, we found that UCHL1 correlates with motor function in PD, with higher levels seen in later disease stages. These findings will be validated in longitudinal studies.

## INTRODUCTION

Parkinson’s disease (PD) is characterized by Lewy bodies and Lewy neurites containing α-synuclein and ubiquitin aggregates, their co-occurrence possibly linked to a failure of the ubiquitin proteasome system (UPS) or the autophagic pathway [1]. Ubiquitin C-terminal hydrolase L1 (UCHL1) plays an important role in maintenance of nervous system integrity, and *in vitro*, overexpression of UCHL1 has been shown to increase ubiquitin levels, ensuring ubiquitin stability within neurons [2]. A rare gene variant of *UCHL1* has been linked with familial PD and a recent meta-analysis demonstrated moderate evidence of an association between a common variant and sporadic PD [3]. CSF ubiquitin levels have been reported to be lower in PD compared to controls [4], but to our knowledge, plasma UCHL1 levels and their relationship with clinical outcomes in PD has not yet been reported. Additionally, the alpha-synuclein gene (*SNCA*) Rep1 promoter region, a polymorphic microsatellite repeat upstream of the *SNCA* transcription start site that enhances *SNCA* transcription and α-synuclein expression [5], has been shown in multiple cohorts to be a risk factor for sporadic PD in carriers of long Rep1 alleles [6, 7]. Long Rep1 allele carriers have been associated with greater risk for motor and cognitive decline in PD [8–10], but no study has yet investigated *SNCA* Rep1 length with UCHL1. We aimed to address these gaps by measuring plasma UCHL1 using an ultrasensitive method in PD and investigate their association with cognitive, motor and disability scores, as well as with *SNCA* Rep1 allele lengths.

## RESULTS

A total of 291 subjects were included (49 controls and 242 PD patients). Their demographic and clinical details are listed in Table 1. Plasma UCHL1 levels were similar between genders in both PD and controls. There was significant association between UCHL1 and age (rho= 0.295, *p*< 0.001), and with disease duration (rho= 0.272, *p*< 0.001) in the PD group, but not in healthy controls.

**Table 1:** Demographic and clinical characteristics of all subjects.

	**HC**	**PD**	***p***
**Subjects, n**	49	242	
**Age, y**	62.6 ± 7.2	65.2 ± 9.7	0.067
**Male, n (%)**	19 (39)	148 (61)	0.004
**Age at onset, y**		61.4 ± 10.0	
**Disease duration, y**		3.8 ± 4.0	
**MDS-UPDRS Part II: ADLs**		10.1 ± 7.5	
**MDS-UPDRS Part III: Motor**		26.4 ± 12.9	
**H&Y stage (n per stage) 1/1.5/2/2.5/3/4**		17/10/180/16/10/9	
**MMSE**	28.7 ± 1.4	26.3 ± 3.3	<0.001
**MoCA**	28.1 ± 0.9	25.7 ± 3.9	<0.001

Data are expressed as mean ± standard deviation or percentages (%). Comparison was performed using two-sided two-sample *t-*tests for continuous variables; Chi-squared tests for categorical variables.

Abbreviations: HC= Healthy Control; PD = Parkinson’s disease; MDS-UPDRS = Movement Disorder Society Unified Parkinson’s Disease Rating Scale; ADL = Activities of Daily Living; H&Y stage = Hoehn and Yahr stage; MMSE = Mini Mental State Examination, MoCA = Montreal Cognitive Assessment.

**Figure 1:**
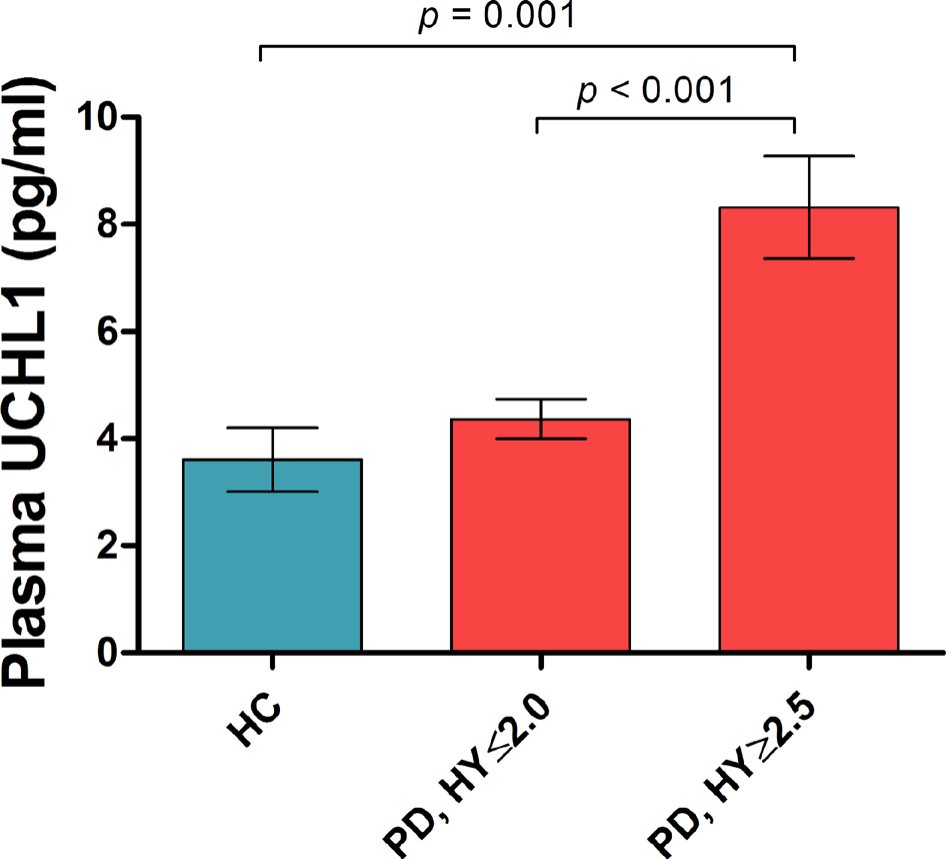
**Plasma UCHL1 levels according to Hoehn-Yahr stage.** Values are mean ± SEM. Plasma UCHL1 levels were significantly higher in PD patients at H&Y stage >2.5 (n=35), compared to controls (n=49) and mild PD patients at H&Y stage ≤2.0 (n=207), controlled for age, gender and Bonferroni method. Abbreviation: HC = Health Control; PD = Parkinson’s disease; HY = Hoehn-Yahr stage.

Plasma UCHL1 levels were significantly different across Hoehn and Yahr (H&Y) stages in PD (p<0.001, adjusted for age and gender; Figure 1). Plasma UCHL1 levels across each H&Y stage are shown in Supplementary Figure 1. Plasma UCHL1 levels were significantly higher in PD patients at H&Y stage >2, compared to controls (8.32 vs 3.61 pg/ml, *p*= 0.001) and mild PD patients at H&Y stage ≤2 (8.32 vs 4.36 pg/ml, *p*< 0.001), adjusted for age, gender and multiple comparisons. When disease duration was added as a covariate, plasma UCHL1 was significantly higher in H&Y stage >2 compared to H&Y ≤2 (p= 0.003). Additionally, the results remained significant when MMSE was added as a covariate. Higher UCHL1 levels correlated with higher MDS-UPDRS Part III motor scores in PD across all stages (*β*= 3.87, *p*= 0.028, controlling for age, gender and disease duration, Table 2), but not with MMSE or MoCA scores. In healthy controls, no significant association was seen between UCHL1 and MMSE (β= -0.901, p= 0.092) or MoCA (*β*= -0.280, p= 0.495) scores, controlling for age and gender.

**Table 2:** Multivariable analysis on plasma UCHL1 levels with cognitive and motor outcomes in PD patients.

**Variables**	**MMSE^a^**		**MoCA**		**MDS-UPDRS Part III (Motor)**	
	**β^b^ (95% CI)**	***p***	**β (95% CI)**	***p***	**β (95% CI)**	***p***
Plasma UCHL1	0.293 (-0.750, 1.335)	0.581	-1.028 (-2.602, 0.546)	0.199	3.871 (0.427, 7.315)	0.028

^a^ MMSE, MoCA or MDS-UPDRS Part III (Motor) scores of PD patients are the outcome variables. ^b^ β is beta coefficient of the multivariable linear regression model adjusted for age, gender and disease duration.

Abbreviation: PD = Parkinson’s disease; MDS-UPDRS = movement disorder society unified Parkinson’s disease rating scale; MMSE = mini mental state examination; MoCA= Montreal Cognitive Assessment.

**Figure 2:**
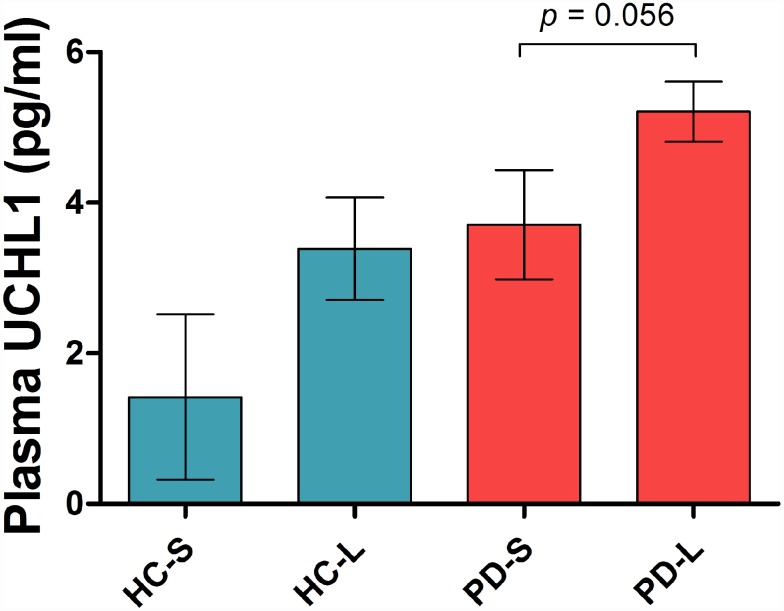
**Plasma UCHL1 levels according to *SNCA* Rep1 genotype.** Values are mean ± SEM. In PD patients, higher UCHL1 levels in carriers of long *SNCA* Rep1 alleles compared to carriers of short alleles, adjusted for age, gender and disease duration. Abbreviation: HC = Health Control; PD = Parkinson’s disease; S = Rep1 short allele; L = Rep1 long allele.

Patients were stratified according to their *SNCA* Rep1 allele length and investigated for the effect of Rep1 genotype on plasma UCHL1. There were 83% long *SNCA* Rep1 and 17% short carriers in the PD group; while there were 77% long and 23% short carriers in the healthy control group. In the PD group, there was a near-significant trend towards higher UCHL1 levels in carriers of long *SNCA* Rep1 alleles vs short alleles (5.21 vs 3.71 pg/ml, *p*= 0.056, Figure 2), while a non-significant trend was seen in healthy controls carrying long Rep1 vs short alleles (3.39 vs 1.42 pg/ml, *p*= 0.207), adjusted for age and gender, and disease duration in the PD group.

## DISCUSSION

Overall, we found that plasma UCHL1 levels were significantly higher in PD patients at more moderate stages of disease (H&Y >2), compared to both PD patients at milder stages (H&Y ≤2) and to healthy controls. Furthermore, higher plasma UCHL1 levels were associated with worse MDS-UPDRS motor scores across all disease stages, but not with global cognitive function in PD. CSF UCHL1 levels have been reported to be lower in PD patients compared to controls [4], with the authors hypothesizing that the reduction of UCHL1 in CSF occurs as a consequence of intraneuronal accumulation and deposition of UCHL1 associated with α-synuclein in cortical Lewy bodies. This hypothesis was supported by the fact that they found the strongest association between UCHL1 and α-synuclein in the PD group. Additionally, their correlation analysis showed that CSF UCHL1 concentrations positively correlated with H&Y stages and age in PD, similar to our findings. However, UCHL1 levels in CSF have been conflicting, with results from proteomic profiling studies done by mass spectrometry reporting increased UCHL1 in CSF of Lewy body dementia (LBD) and PD patients compared to healthy controls [11]. The direct function of UCHL1 remains unclear and a wide array of alternative functions has been suggested [12]. We hypothesize that UCHL1 levels may increase in moderate stages of disease possibly as part of compensatory mechanisms in response to greater α-synuclein burden [13], given that phosphorylated α-synuclein levels are known to correlate with disease severity in Lewy body disorders [14]. In Alzheimer’s disease (AD) transgenic mice, *over*expression of UCHL1 has been shown to reduce Aβ production, inhibit neuritic plaque formation and improve memory deficits [15], while in PD, suppression of UCHL1 activity *in vitro* has been shown in non-transgenic neurons to increase accumulation of presynaptic α-synuclein [16]. Post-mortem studies have demonstrated reduced UCHL1 mRNA expression and protein levels in both the cortex and substantia nigra in cases with Lewy body pathology [17].

Interestingly, we found that UCHL1 levels were higher in both PD and control carriers of long *SNCA* Rep1 alleles compared to short allele carriers. These results were not surprising given the strong biological plausibility that carriers of longer Rep1 alleles may possess greater α-synuclein burden through increased *SNCA* gene expression, with homozygosity for the expanded/longer Rep1 allele potentially mimicking *SNCA* locus multiplication [5]. α-synuclein levels in blood and human post-mortem brain tissue were reported to be lower in carriers of shorter vs longer Rep 1 alleles, providing pathological evidence that α- synuclein levels are influenced by variability in the *SNCA* promoter region [18]. Although these results did not reach statistical significance, there was a near- significant trend in the PD group (p=0.056) that warrants further investigation in a larger sample size.

UCHL1 was originally identified as a neuronal cytoplasmic protein that accounts for nearly 2% of total brain proteins [19, 20]. Later studies have reported UCHL1 expression in cells of the diffuse neuroendocrine system, testis and certain tumours. The source of UCHL1 in the peripheral blood however, remains unclear as to our knowledge, there have not been reports on UCHL1 expression in red blood cells, platelets or leukocytes. We hypothesized that UCHL1 was likely released from CSF into the peripheral circulation via impaired blood-brain barrier, which is involved in the course of neurodegenerative diseases [21, 22]. UCHL1 has both hydrolase and ligase activity in the ubiquitin-proteasome pathway and previous studies have shown that mutations in *UCHL1* and oxidative modifications of the UCHL1 protein affect enzymatic activity, thereby contributing to neurodegeneration [23–26]. Aberrant ubiquitin hydrolase and/or ligase activities can lead to dysfunction of the proteasome proteolytic system, accumulation of damaged proteins and formation of protein (α-synuclein) aggregates, which are pathological hallmarks of PD. However, no study to our knowledge has investigated the function of secreted UCHL1 in sporadic PD. Increased extracellular UCHL1 may be a compensatory mechanism in response to increased α-synuclein burden (given that higher CSF UCHL1 levels correlated with higher α-synuclein) or may reflect reduced levels of functional UCHL1 in the neurons, leading to a dysfunction in ubiquitin-proteasome system.

In summary, we used ultrasensitive single molecule technology to show that plasma UCHL1 levels are significantly higher in PD patients at more moderate stages of disease than in both mild PD patients and healthy controls. UCHL1 levels significantly correlated with motor severity, but not global cognition. To our knowledge, this will be the first study reporting UCHL1 levels in blood samples from PD patients, as well as the first study investigating the relationship between *SNCA* Rep1 promoter variability and UCHL1. Although the study included a substantial number of PD patients, we acknowledge the comparatively smaller sample size of healthy controls. Future studies with a larger cohort of PD patients and healthy controls are needed to confirm our findings. Our preliminary findings provide impetus for further validation in longitudinal cohorts to determine if UCHL1 correlates with disease progression. If confirmed, modulation and possibly overexpression of UCHL1 activity could serve as a therapeutic tool in enhancing the autophagy pathway and inducing clearance of α-synuclein aggregates in PD. Studies to assess its potential utility as a predictive biomarker and as a novel therapeutic target in PD are warranted.

## MATERIALS AND METHODS

### Clinical recruitment

Subjects were recruited from the movement disorder clinics at the National Neuroscience Institute, Singapore, between November 2014 and February 2018. All PD patients fulfilled National Institute of Neurological Disorders and Stroke (NINDS) criteria [27] for the diagnosis of PD. Disease staging was determined with the Hoehn and Yahr (H&Y) rating scale [28] and motor severity with the Movement Disorders Society Unified Parkinson’s Disease Rating Scale part III (MDS-UPDRS) [29]. UPDRS scores were obtained with nearly all patients in the “on” state. Global cognition was measured using the Mini-Mental State Examination (MMSE) [30] and Montreal Cognitive Assessment (MoCA) tool [31]. Healthy controls were recruited from the community and were free of significant neurological, psychiatric or systemic disease as examined by a trained neurologist.

### Standard protocol approvals, registrations, and patient consents

Ethics approval was obtained from the SingHealth Centralised Institutional Review Board, and all participants provided informed written consent prior to data collection.

### Plasma UCHL1 measurement

EDTA blood was collected from each subject by venipuncture and centrifuged at 1,500 g for 15 minutes within an hour after blood collection. Plasma was aliquoted and stored at −80°C until further analysis. UCHL1 levels were measured using ultrasensitive single molecule array (Simoa) Human Ubiquitin C-terminal hydrolase L1 assay on a Simoa HD-1 Analyzer (Quanterix, MA), according to the manufacturer’s protocol. This assay was developed with two monoclonal antibodies, from Banyan Biomarkers, specific to the UCHL1 protein. Two QC samples, prepared from the kit’s Calibrator Concentrate and measured in each run, were both in the expected range. Mean coefficient of variation (CV) of UCHL1 measurement in QC samples was <5% and plasma samples was <20%.

### *SNCA* Rep1 genotyping

Genomic DNA was extracted from peripheral blood with QIAamp® DNA Blood Maxi Kit (Qiagen) according to the manufacturer’s protocol. Fragment length analysis of *SNCA* Rep1 was performed using polymerase chain reaction (PCR) with Q5 High-Fidelity DNA Polymerase (New England Biolabs), primers Rep1-F (5’FAM CCTGGCATATTTGATTGCAA 3’) and Rep1-R (5’ GACTGGCCCAAGATTAACCA 3’), under the conditions: 98°C 30”, 35 cycles of 98°C 10”, 60°C 30” and 72°C 30”, and 72°C 2’. Repeat sizes of PCR products were determined on the ABI 3500xL Genetic Analyzer using GeneScan 500 ROX standards (Applied Biosystems). Rep1 allele length was coded according to previous methods described by Farrer et al. [6], and subjects carrying allele lengths coded as 1 were classified as “short” carriers, while subjects carrying allele lengths coded as 2 or 3 were classified as “long” carriers.

### Statistical analysis

Differences in demographic data between PD and controls were assessed by two-tailed unpaired t-test for continuous variables, and Chi-square test for categorical variables. Correlations between clinical variables and plasma UCHL1 levels were calculated using Spearman’s correlation coefficient. Plasma UCHL1 levels were log-transformed to achieve a normal distribution for subsequent analysis. For group-wise comparisons of UCHL1, we used univariate general linear models controlling for possible confounders (e.g., age, gender, disease duration) and pair-wise comparisons with Bonferroni *post-hoc* test. Associations between UCHL1 levels and cognitive and motor variables were assessed by multivariable linear regression analysis adjusted for possible confounders. P ≤ 0.05 was considered statistically significant. All statistics were performed using SPSS version 22 (IBM).

## Supplementary Material

Supplementary Figure 1
