# Supplementary material for: Plasma ubiquitin C-terminal hydrolase L1 levels reflect disease stage and motor severity in Parkinson’s disease
Source: Aging (Albany NY). 2020 Jan 13;12(2):1488–95. doi: 10.18632/aging.102695 (PMC7053593; doi:10.18632/aging.102695)
Supplement: Supplementary Figure 1 [file aging-12-102695-s001..pdf]

SUPPLEMENTARY FIGURE

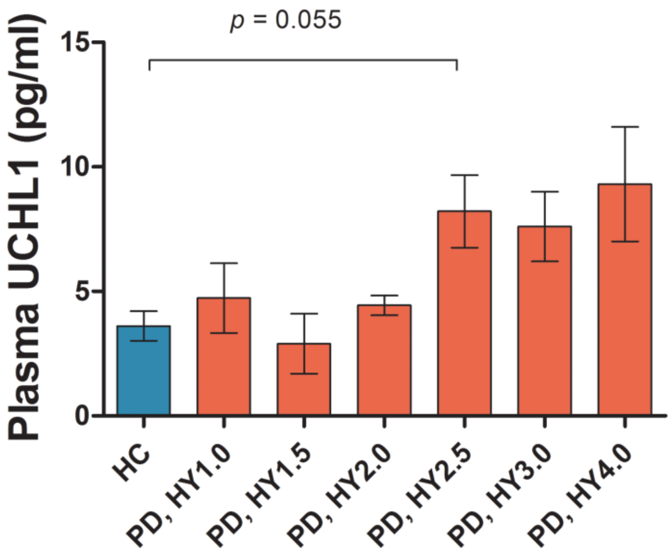

Supplementary Figure 1. Plasma UCHL1 levels according to Hoehn-Yahr stage. Values are mean ± SEM. Plasma UCHL1 levels were significantly higher in PD patients at H&Y stage 2.5, compared to HC, controlled for age, gender and Bonferroni method. Abbreviation: HC = Health Control; PD = Parkinson's disease; HY = Hoehn-Yahr stage**.**
